# Solubility, Antioxidation, and Oral Bioavailability Improvement of Mangiferin Microparticles Prepared Using the Supercritical Antisolvent Method

**DOI:** 10.3390/pharmaceutics12020090

**Published:** 2020-01-22

**Authors:** Mengran Liu, Yankun Liu, Yunlong Ge, Zhaoliang Zhong, Zijian Wang, Tong Wu, Xiuhua Zhao, Yuangang Zu

**Affiliations:** 1 College of Chemistry, Chemical Engineering and Resource Utilization, Northeast Forestry University, Harbin 150040, Heilongjiang, China; forestryliu@163.com (M.L.); zhongzhaoliang999@163.com (Z.Z.); wzj_234313256@163.com (Z.W.); 2 Key Laboratory of Forest Plant Ecology, Northeast Forestry University, Ministry of Education, Harbin 150040, Heilongjiang, China; 3 School of Pharmacy, Heilongjiang University of Chinese Medicine, Harbin 150040, Heilongjiang, China; lyk9911@163.com; 4 Medical College, Harbin Vocational & Technical College, Harbin 150081, Heilongjiang, China; wo-geyunlong@163.com; 5 School of Architecture, Harbin Institute of Technology, Harbin 150001, Heilongjiang, China; wutong_hit@163.com

**Keywords:** mangiferin, mangiferin microparticles, solubility, bioavailability, antioxidation

## Abstract

In view of the poor water solubility and low oral bioavailability of mangiferin (MG), in this study, the supercritical antisolvent (SAS) technology was used to prepare mangiferin microparticles (MG MPs) with *N*,*N*-dimethylformamide (DMF) as solvent and carbon dioxide as antisolvent, so as to improve its water solubility, antioxidant capacity and oral bioavailability. Four factors affecting the solubility of the MG MPs were investigated by orthogonal design (OAD), including precipitation pressure, precipitation temperature, MG concentration and feeding speed, and the optimal preparation conditions were determined by range and variance analysis (ANOVA). Under the optimal conditions, the spherical MG MPs with an average diameter of 532.8 nm was obtained, and the yield of the powder was about 95.3%. Scanning electron microscopy (SEM), fourier transform infrared spectroscopy (FTIR), X-Ray Diffractometry (XRD), differential scanning calorimetry (DSC), and thermal gravimetric (TG) were used to analyze the characteristics of the MG MPs. The results obtained showed that the chemical structure of the MG did not change before and after supercritical crystallization, but its particle size and crystallinity decreased significantly. The MG MPs had a higher solubility, and was about 4.26, 2.1 and 2.5 times than that of free MG in water, artificial gastric juice (AGJ) and artificial intestinal juice (AIJ), respectively. The dissolution rate of the MG MPs were also obviously higher than that of free MG. Furthermore, the bioavailability of the MG MPs in vivo was about 2.07 times higher than that of the free MG, and its antioxidant capacity was also much higher than that of free MG, which was close to vitamin C.

## 1. Introduction

**Figure 1:**
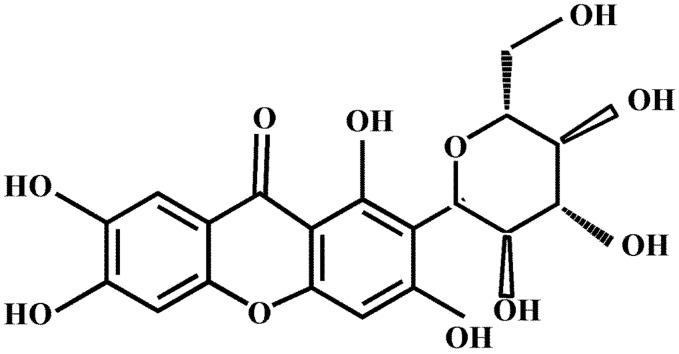
Chemical structure of mangiferin.

Mangiferin (MG, Figure 1), a naturally produced polyphenol molecule possessing four hydroxyl groups, is an efficient antioxidant for free radical chain termination [1], which is mainly distributed in *Anemarrhena asphodeloides* (Liliaceae family), *Mangifera indica* L. (Anacardiaceae family) and *Belamcanda chinensis* L DC (*Belamcanda adans*) [2]. Its molecular formula is C_19_H_18_O_11_, and its molecular weight is 422 [3].

In recent years, it had been found that the MG has many pharmacological activities, such as anti-oxidation, anti-tussive, expectorant, anti-asthmatic, central inhibition, anti-inflammatory, anti-bacterial, anti-virus, anti-tumor, cholagogic and immunomodulatory effects [4,5,6,7,8]. However, the clinical application of the MG was severely limited due to its poor solubility in water, low bioavailability and low efficacy [9]. At present, the structural modification method and some solubilization technologies had been used to improve the water solubility and bioavailability of the MG. For example, Yuan et al. reported that the 3-phenolic hydroxyl of the MG was converted into phenol sodium salt to improve the water solubility, thus promoting its absorption in the gastrointestinal tract, improving its bioavailability and efficacy [10]. Liao et al. Reported that a series of 3,6,7-*o*-trisubstituted MG derivatives were obtained by alkylation of the MG [11]. The preliminary screening results of anti diabetes activity in vitro showed that the inhibition rate of the derivatives on protein tyrosine phosphatase was twice higher than that of the MG at the same concentration. Although the structure modification increased the water solubility of the MG to some extent, there were potential risks of high production cost, low conversion rate and unclear metabolism in vivo. Additionally, in terms of solubilization technologies, some new formulations of the MG have been developed, such as nanoemulsions [12], phospholipid complex [13,14], cyclodextrin inclusion complex [15] and liposome [16,17], and so on. These solubilization methods had improved the dissolution and bioavailability of the MG a certain extent, but there were still problems such as large amount of excipients and low drug loading, which might lead to potential toxicity problems of excipients.

Drug micronization was an effective method to solve the dissolution of insoluble drugs, and the supercritical antisolvent (SAS) technology was a new type of micronization technology developed in recent years, especially suitable for the preparation of micro/nanoparticles of the pure drugs. Due to the high purity and few excipients of the drug micro/nanoparticles obtained, this method has attracted the attention of the pharmaceutical industry. At present, a variety of nanoparticles of the drugs such as paclitaxel [18], griseofulvin [19], ursolic acid [20], atorvastatin calcium [21], and so on, have been successfully prepared by the SAS method, and they showed good water solubility, bioavailability and activities. In addition, it had been reported that the Nano- and microparticles of mangiferin was prepared by a supercritical antisolvent (SAS) process, and the solvent mixtures of acetone with dimethyl sulfoxide was used as the solvent, and only the dissolution characteristics of the nano- and microparticles of mangiferin were discussed preliminarily [22]. However, the objective of the present study was to develop the MG microparticles by using the SAS technology. A single solvent, *N*,*N*-dimethylformamide (DMF), was used as the solvent. The morphology, physical and chemical properties, solubility, dissolution characteristics, antioxidant activity and bioavailability of the MG microparticles were comprehensively investigated. Consequently, this study not only prepared MG microparticles with high water solubility and bioavailability, but also provided important reference value for the development and clinical application of the MG microparticles.

## 2. Materials and Methods

### 2.1. Materials

Mangiferin standard (98%) was purchased from Shanghai Lvyuan Co., Ltd. (Shanghai, China); free mangiferin (97%) was purchased from Xi’an Tianfeng Biotechnology Co., Ltd. (Xian, China); *N*,*N*-dimethylformamide, acetonitrile (HPLC grade), methanol (HPLC grade), ethanol (A.R. grade), NaOH, KH_2_PO_4_, hydrochloric acid, trichloroacetic acid, potassium ferricyanide and ferric chloride were purchased from Tianjin Tianli Chemical Reagent Co., Ltd. (Tianjin, China); high-purity CO_2_ (99.99%) was obtained from Harbin Arctic Gas Co., Ltd. (Harbin, China); deionized water (self-made laboratory); heparin sodium was purchased from Shanghai Huishi biochemical reagent Co., Ltd. (Shanghai, China); SD rats were obtained from Animal Center of the First Affiliated Hospital of Harbin Medical University (Harbin, China); vitamin C was purchased from Hangzhou Jingjiu Chemical Co., Ltd. (Hangzhou, China); 1,1-diphenyl-2-dinitrophenylhydrazine (DPPH) and 2,2′-azino-bis-(3-ethylbenzthiazoline-6-sulfonic acid) (ABTS) were purchased from Sigma (Shanghai, China).

### 2.2. Supercritical Antisolvent (SAS) Device

**Figure 2:**
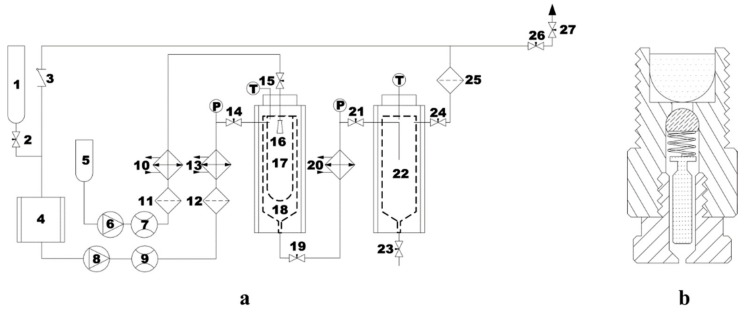
Schematic diagram of the supercritical antisolvent (SAS) device (**a**) and the nozzle (**b**). (1) CO_2_ cylinder; (2, 14, 15, 19, 21, 23, 24, 26, and 27) valves; (3) check valve; (4) CO_2_ cooler; (5) liquid solution supply; (6) liquid pump; (8) CO_2_ pump; (7 and 9) flow meter; (10, 13 and 20) heat exchangers; (11, 12, and 25) filters; (16) nozzle; (17) stainless steel frit vessel of 200 nm; (18) precipitation chamber; (22) gas–liquid separation chamber.

The schematic diagram of supercritical antisolvent (SAS) device is shown in Figure 2a. The device included the precipitation chamber and the gas–liquid separation chamber. Before compression with the liquid pump (8), the CO_2_ was cooled by the cooler (4), and the pressure was controlled by the back pressure regulating valve. After preheating in the heat exchanger (13), CO_2_ entered the precipitation chamber (18). Meanwhile, the solution pump was started, heated, and fed to the precipitation chamber (1000 mL) through stainless steel nozzle (16). Figure 2b showed a close-up of the nozzle details, the nozzle was the inner diameter of the laser drilling hole (inner diameter 150 μm), which was located at the top of the precipitation chamber. The stainless steel frit vessel with a pore diameter of 200 nm (17) was put into the precipitation chamber to collect micropowders and let the DMF–CO_2_ organic solvent mixture pass through. The flow rate of the mixture leaving the crystallization kettle was controlled by the valve (21) between the precipitation chamber and the gas–liquid separation chamber (22), wherein the mixture was separated from the organic solvent by reducing pressure (pressure < 5 MPa).

### 2.3. Preparation of the MG MPs

Firstly, CO_2_ was delivered to the precipitation chamber until the required pressure was reached, and the flow rate of CO_2_ was 21 kg/h. Then, DMF was delivered to the precipitation chamber through the feed pump to obtain the steady-state composition conditions during the extraction of the MG. At this time, the DMF stops flowing and MG/DMF liquid solution was delivered through the nozzle. At this time, the DMF stops flowing and MG/DMF liquid solution was delivered through the nozzle. After stopping the feed pump, supercritical CO_2_ continued to flow for 50 min to wash the residual liquid in the precipitation chamber. The pressure in the precipitation chamber was then gradually reduced to atmospheric pressure. Finally, the MG MPs powders was extracted from the precipitation chamber for further characterization and analysis.

### 2.4. Optimization of Preparation Process of the MG MPs

L_16_ (4^5^) orthogonal test was designed to optimize the operation conditions of the MG MPs by SAS technology. As shown in Table 1, the SAS experiment was conducted with four factors and four levels, namely, precipitation pressure (10, 15, 20, 25 MPa); precipitation temperature (35, 43, 51, 59 °C); MG concentration (5, 23, 41, 59 mg/mL); feeding speed (4, 7, 10, 13 mL/min). The value range of each factor was based on the results of previous single factor experiment, and the saturated solubility of the MG MPs in water (mg/mL) was the response value.

### 2.5. Characterization of the MG MPs

#### 2.5.1. Particle Size Detection

The average particle size of the MG MPs powders was measured by dynamic laser light scattering technique (ZetaPALS, Brookhaven, Holtsville, NY, USA). Before measurement, the MG MPs (3 mg) were diluted properly by deionizer water (3 mL). The measurements were done in triplicate.

#### 2.5.2. Scanning Electron Microscopy (SEM)

MG MPs powders and free MG were fixed on SEM stubs by double-sided conductive adhesive, and then made electrically conductive by sputter-coating with gold using the ion sputtering coating machine. The particle morphology of the MG MPs powders and the free MG was observed by a SEM (FEI, Eindhoven, Netherlands).

#### 2.5.3. Fourier Transform Infrared Spectroscopy (FTIR)

The FTIR spectrum of the MG MPs powders and the free MG in the wavenumber range of 400–4000 cm^−1^ were examined by a FT-IR spectrophotometer (SHIMADZU, Kyoto, Japan). Each dry sample was accurately weighed 2 mg, and mixed with KBr of 200 mg respectively, and then grinded into fine powders and pressed into transparent slices for analysis.

#### 2.5.4. Differential Scanning Calorimetry (DSC) and Thermal Gravimetric (TG)

The heating stability of the MG MPs powders and the free MG was studied by DSC (Setaram, Lyon, France), the samples were operated at a heating rate of 10 °C/min from 30 °C to 370 °C.

The thermal gravimetric of the MG MPs powders and the free MG was researched using a thermo-gravimetrical 200 Analyzer (PerkinElmer, Waltham, MA, USA) at a heating rate of 10 °C/min in a temperature range of 30–370 °C, and the whole process was carried out under dynamic nitrogen atmosphere.

#### 2.5.5. X-Ray Diffraction (XRD)

The crystal structure of the MG MPs powders and the free MG was analyzed by an X-ray diffractometer (Rigaku Corporation, Tokyo, Japan). The diffraction angle range was 5–60°; The voltage and current were 40 kV and 30 Ma, respectively; The scanning rate was 5°/min.

### 2.6. In Vitro Dissolution Study of the MG MPs

#### 2.6.1. Preparation of Artificial Gastric Juice and Artificial Intestinal Juice

In this study, artificial gastric juice (AGJ) and artificial intestinal juice (AIJ) were selected as dissolution media, which were prepared as follows: The AGJ was obtained by mixing 3.84 mL hydrochloric acid, 800 mL water, 10 g pepsin and 4 g Tween-80, then adding water to a constant volume of 1000 mL. In addition, KH_2_PO_4_ 6.8g was taken and dissolved with 500 mL of water, and then the pH value was adjusted to 6.8 with 0.1 mol/L NaOH solution. Another 10 g trypsin was taken and dissolved by adding appropriate amount of water. After mixing the two liquids, the AIJ was obtained by adding water to a constant volume of 1000 mL.

#### 2.6.2. Determination of Saturated Solubility

The MG MPs (20 mg) and the free MG (20 mg) were put into small vials containing 2 mL of water, AGJ and AIJ, respectively and sealed, and then all the vials were put in the water bath of 37 °C, 40 rpm/min. After 48 h, the samples were taken out and centrifuged at 8000 rpm for 10 min, and then filtered through a 0.22 μm membrane filter. The supernatant obtained was used for HPLC detection to determine MG concentration. The HPLC detection was implemented by a Waters 1525-2489 high performance liquid chromatograph (Waters Corporation, Milford, MA, USA) consisting of a pump (Waters 1525 binary) and UV detector (Waters 2489 Tunable Absorbance Detector), which was equipped with a Diamonsil C18 reverse-phase column (4.6 × 250 mm, 5 μm, DIKMA, Beijing, China). The mobile phase, composed of acetonitrile and 0.1% phosphoric acid, was delivered at 1 mL/min. The drug was detected at 325 nm and the injection volume was 10 μL. The linear regression equation *y* = 13,866,374.4100*x* + 50,262.5833 (*R*^2^ = 0.9998) was obtained with concentration of MG standard solution as abscissa and the absorbency as the *y*-coordinate.

#### 2.6.3. In Vitro Dissolution Test

The in vitro dissolution of the MG MPs and the free MG was investigated in the two media mentioned above. Dissolution tests were consistent with the sink conditions and were carried out at 37.0 ± 0.5 °C at a rotation speed of 100 rpm. Samples equivalent to 8.2 mg of MG were weighed and dispersed in a 250 mL beaker containing 200 mL of dissolution media. The samples (1 mL) were withdrawn at 0.083, 0.167, 0.25, 0.33, 0.5, 0.75, 1.0, 1.5, 2.0, 3.0, 4.0, 5.0, 6.0, 8.0, 10.0, 12.0, and 24.0 h, and then supplemented with the same volume of dissolution medium. The samples obtained were centrifuged at 8000 rpm for 10 min and then filtered through 0.22 μm membrane filters to separate the excess drug. The final supernatant was determined by the HPLC detection. Then the cumulative drug release was calculated according to the Equations (1)–(3).

(1) $$C_{1}^{\prime }=C_{1}$$

(2) $$C_{i+1}^{\prime }=C_{i+1}-\frac{\left({V-V}_{i}\right)C_{i}}{V_{}}$$

(3) $$Q_{i}=\frac{\sum_{i=1}^{i}C_{i}^{\prime }\overset{}{V}}{M}\times 100\%$$

$C_{i}^{\prime }$: MG concentration in each time interval (mg/mL);

$\overset{}{V}$: Total volume of dissolution medium (mL);

Vi: Volume of each sample (mL);

M: Total MG input (mg);

$Q_{i}$: Cumulative release percentage (%) at each sampling point (%).

### 2.7. Evaluation of Antioxidant Activity In Vitro of the MG MPs

#### 2.7.1. Measurement of DPPH Radical-Scavenging Activity

We accurately weighed 8 mg of the MG MPs, 8 mg of the free MG, and 8 mg of vitamin C and separately added them into three vials containing 20 mL of deionized water, then the samples were treated with ultrasound for 10 min. Then the suspensions of each sample were centrifuged for 10 min with 8000 rpm/min, and the supernate obtained was diluted into different concentrations, namely 0.1 mg/mL, 0.05 mg/mL, 0.025 mg/mL, 0.0125 mg/mL, 0.00625 mg/mL and 0.00313 mg/mL (calculated by the initial suspension). Each sample (1.5 mL) with different concentration were mixed with 1.5 mL of ethanol DPPH solution (0.3 mmol/L) in numbered tubes, after mixed evenly, the reaction tubes were placed at room temperature in the dark. After 30 min, the absorbance of the samples was measured at 517 nm [23]. The experiment was repeated three times and the average value was taken. The ability to scavenge DPPH radical was calculated by the following Equation:

DPPH radical-scavenging rate (%) = [(*Ac* − *Ai*)/*Ac*] × 100% (4)

where *Ac* is the absorbance of the control, and *Ai* is the absorbance of the sample.

#### 2.7.2. Measurement of ABTS Radical-Scavenging Activity

We accurately weighed 8 mg of the MG MPs, 8 mg of the free MG, and 8 mg of vitamin C and separately added them into three vials containing 20 mL of deionized water, then the samples were treated with ultrasound for 10 min. Then the suspensions of each sample were centrifuged for 10 min with 8000 rpm/min, and the supernate obtained was diluted into different concentrations, namely 0.40 mg/mL, 0.20 mg/mL, 0.10 mg/mL, 0.05 mg/mL, 0.025 mg/mL, 0.0125 mg/mL, 0.00625 mg/mL and 0.00313 mg/mL (calculated by the initial suspension). Each sample (0.2 mL) with different concentration were mixed with 4 mL of ABTS solution (7 mmol/L) in numbered tubes, after mixed evenly, the reaction tubes were placed at room temperature in the dark. After 30 min, the absorbance of the samples was measured at 734 nm [24]. The experiment was repeated three times and the average value was taken. The ability to scavenge ABTS radical was calculated by the following Equation:

ABTS radical-scavenging rate (%) = [(*Ao* − *A*)/*Ao*] ×100% (5)

where *Ao* is the absorbance of the control, and *A* is the absorbance of the sample.

#### 2.7.3. Measurement of Reducing Power

We accurately weighed 8 mg of the MG MPs, 8 mg of the free MG and 8 mg of vitamin C and separately added them into three vials containing 20 mL of deionized water, then the samples were treated with ultrasound for 10 min. Then the suspensions of each sample were centrifuged for 10 min with 8000 rpm/min, and the supernate obtained was diluted into different concentrations, namely 0.40 mg/mL, 0.20 mg/mL, 0.10 mg/mL, 0.05 mg/mL, 0.025 mg/mL, 0.0125 mg/mL, 0.00625 mg/mL and 0.00313 mg/mL (calculated by the initial suspension). 2ml of each sample with different concentration was mixed with 0.2 mol/L of phosphate buffer (pH 6.6, 2 mL) and 1% potassium ferricyanide (2 mL). The mixture was kept at 50 °C for 20 min, then 2 ml of 10% TCA was added to stop the reaction. Then the mixture was centrifuged at 3500 r/min for 10 min, and the supernatant obtained was mixed with distilled water and 0.1% FeCl_3_ according to volume ratio of 1:1:0.2 (*v*/*v*/*v*). The absorbance of each sample at 700 nm was measured after 10 min [25]. The higher the absorption value was, the stronger the reducing power was. The experiment was repeated three times and the average value was taken.

### 2.8. Bioavailability Test in Rats

Twelve female SD rats weighing between 200–250 g were randomly divided into two groups, six rats in each group. The experimental protocols were approved by the Institutional Animal Care and Use Committee of Harbin Medical University (approval No. HMUIRB-2008-06) on 23 June 2006. Before the experiment, all rats were fasted for 12 h with free access to water. Then, the rats were administrated with the MG MPs and the free MG by gavage at the doses of 100 mg/kg, respectively. After oral administration, the rats were anesthetized and blood samples obtained from the retro-orbital plexus at 0.083, 0.167, 0.25, 0.33, 0.5, 0.75, 1.0, 1.5, 2.0, 3.0, 4.0, 5.0, 6.0, 8.0, 10.0, 12.0 and 24.0 h, respectively, were put in the centrifuge tubes with 1% heparin sodium. The mixture of heparin sodium and blood was made evenly by shaking gently, and was then centrifuged at 3000 rpm/min for 10 min. The plasma samples were taken out and stored in a refrigerator at 4 °C.

Plasma samples of 0.2 mL and 0.8 mL of methanol were added in 1.5 mL centrifuge tube and vibrated for 3 min by a vortex mixer. After mixing evenly, the mixture was centrifuged for 10 min at 12,000 r/min. The supernate was taken out, and the blood sample was again extracted under the same conditions by the methanol. Finally, the extraction obtained was dried under nitrogen, and then dissolved with 100 μL methanol for analysis by the HPLC system.

## 3. Results and Discussion

### 3.1. Optimization Study

In this experiment, the MG MPs powders was successfully prepared by the SAS technology. Through the L_16_ (4^5^) orthogonal test design, the influence of four factors, including precipitation pressure, precipitation temperature, MG concentration and feeding speed, on the saturated solubility of the MG MPs in water was investigated. The experimental design and response values were shown in Table 2. The ANONA analysis is shown in Table 3.

According to the R value, it could be concluded from Table 2 that the influence of each factor on the saturated solubility of the MG in the order of A > C > B > D. Therefore, when the precipitation pressure, precipitation temperature, MG concentration and feeding speed were 20 MPa, 51 °C, 23 mg/mL and 10 mL/min, respectively, the saturated solubility of the MG MPs was the highest, about 0.6013 mg/mL, and its average particle size and variation coefficient were about 532.8 nm and 2.01%, respectively. In order to confirm the validity of the optimization procedure, the parallel test was carried out for three times under the optimal conditions, and the solubility of the MG MPs was about 0.6413 mg/mL. Thus, the result of the orthogonal test was almost consistent with the actual one, and the model was reliable.

### 3.2. Effect of Preparation Conditions on the Saturated Solubility of the MG MPs

**Figure 3:**
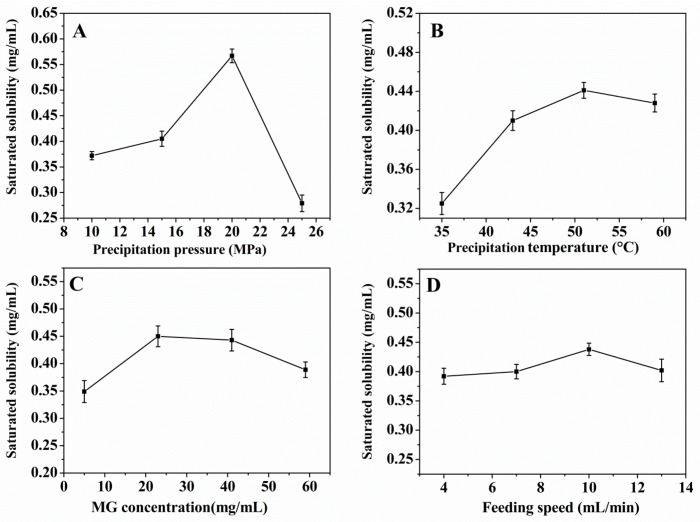
The effect of each factor on the saturated solubility of the mangiferin microparticles (MG MPs). (**A**) Precipitation pressure; (**B**) precipitation temperature; (**C**) MG concentration; (**D**) feeding speed.

The influence of different preparation conditions on the saturated solubility of the MG MPs was shown in Figure 3. From Figure 3A, it can be seen that when the precipitation pressure increased from 10 to 20 MPa, the saturated solubility of the MG MPs increased significantly. When the precipitation pressure was 20 MPa, the saturated solubility of the MG MPs reached the maximum value. But when the precipitation pressure was higher than 20 MPa, the saturated solubility of the MG MPs began to decrease; From the Figure 3B, when the precipitation temperature increased from 35 to 59 °C, the saturated solubility of the MG MPs first increased significantly and then decreased slightly. When the precipitation temperature was 51 °C, the saturated solubility of the MG MPs reached the maximum value; In the Figure 3C, with the MG concentration increasing from 5 to 23 mg/mL, the saturated solubility of the MG MPs began to increase. But when the MG concentration was more than 23, the saturated solubility of the MG MPs decreased gradually. When the MG concentration was 23, the saturated solubility of the MG MPs was the highest. In addition, from Figure 3D, it can be seen that the saturated solubility of the MG MPs increased first and then decreased with increasing the feeding speed from 4 to 13 mL/min. When the feeding speed was 10 mL/min, the saturated solubility of the MG MPs reached the maximum value.

### 3.3. Characterization of the MG MPs

#### 3.3.1. Morphology and Particle Size

**Figure 4:**
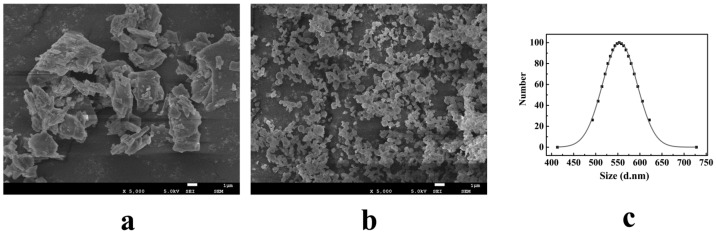
SEM images and normal distribution curve of samples. (**a**) SEM image of the free MG; (**b**) SEM image of the MG MPs; (**c**) normal distribution curve of the MG MPs powder.

Figure 4a shows the SEM image of the free MG. It could be seen from the image that the free MG was irregular long flake shape, and its particle size was greater than 10 μm; Figure 4b shows the SEM image of the MG MPs. It could be seen from the image that the particle size and the particle morphology of the MG MPs prepared by the SAS method have been significantly improved, and its morphology was nearly spherical and homogeneously distributed around 0.4–0.7 μm, and was significantly smaller and more uniform than the free MG.

In addition, the measurement result of particle size of the MG MPs is shown in Figure 4c. From the result, it could be seen that the particle size of the MG MPs was about 532.8 nm, which was almost consistent with the SEM results. From the above mentioned, therefore, the MG MPs prepared by the SAS method had good morphology and particle size, which was conducive to the improvement of water solubility and bioavailability of the MG.

#### 3.3.2. FTIR Results

**Figure 5:**
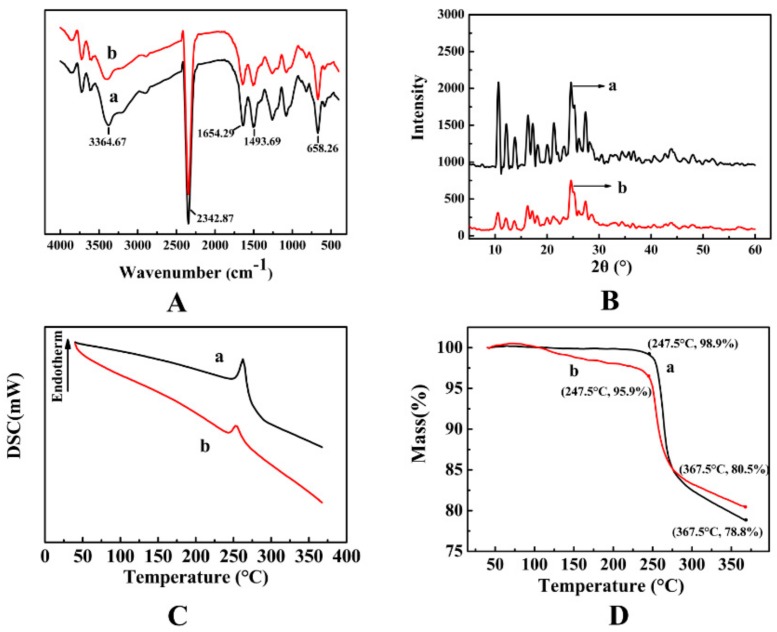
FTIR results (**A**) (Wavenumber: 400–4000 cm^−1^), XRD results (**B**) (2θ = 5–60°), DSC results (**C**) (Temperature range: 30–370 °C) and TG results (**D**) (Temperature range: 30–370 °C) of the samples. (a) the free MG; (b) the MG MPs.

The MG MPs were further analyzed by FT-IR spectroscopy. The FTIR spectra of the free MG and the MG MPs from 4000 to 400 cm^−1^ are shown in Figure 5A. From the Figure 5A, there was no significant difference between the MG MPs (a) and the free MG (b), both of which showed vibration characteristic peaks at 3364.67, 2342.87, 1654.29, 1493.69, 658.26 cm^−1^, etc., which illustrated that the chemical structure of the MG MPs prepared by the SAS technology had not changed.

#### 3.3.3. XRD Results

The degree of crystallinity of the free MG and the MG MPs was evaluated by XRD analysis. The XRD results of the free MG and the MG MPs were shown in Figure 5B. From the Figure 5B, the free MG had obvious diffraction peaks at 2θ = 10.66°, 12.11°, 13.78°, 16.35°, 17.23°, 21.25° and 24.49°, while, the MG MPs has relatively weak diffraction peak at the same position and some diffraction peaks had disappeared, which illustrated that the crystallinity of the MG MPs was obviously lower than that of the free MG, which was conducive to the increase of the solubility and bioavailability of the MG.

#### 3.3.4. DSC and TG Results

DSC analysis results of the free MG and the MG MPs was shown in the Figure 5C. It could be seen from the figure that the free MG (b) had an endothermic peak melting at 260 °C, which was the melting point of the MG, indicating that the free MG existed in crystal form. However, the MG MPs (a) had a relatively weak endothermic peak at 260 °C, which indicated that the crystallinity of the MG MPs was obviously reduced, which was consistent with XRD results.

TG analysis results of the free MG and the MG MPs was shown in the Figure 5D. The figure showed that the MG MPs (a) and the free MG (b) began to lose weight from 100 to 247.5 °C, with the weight loss of about 4.1% and 1.1%, respectively, while they showed significantly weight losses from 247.5 °C, with a weight loss rate of 19.5% and 21.2%, respectively. The results showed that the weight loss of the MG MPs was slightly higher than that of the free MG, which might be due to the smaller particle size and larger specific surface area of the MG MPs, which makes it more easily evaporated and rapidly decomposed.

### 3.4. In Vitro Dissolution Results of the MG MPs

#### 3.4.1. Determination Results of Saturated Solubility

**Figure 6:**
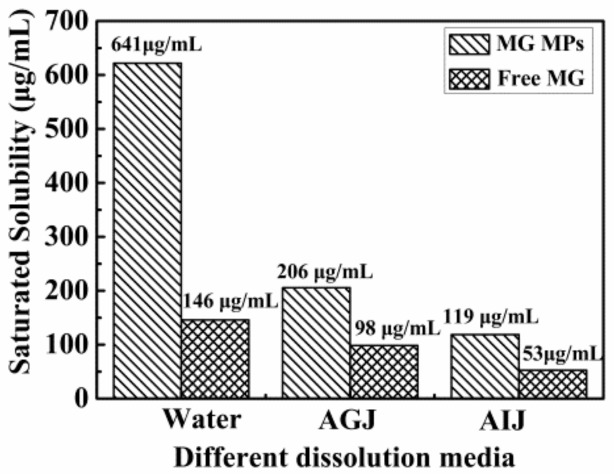
The saturated solubility of the MG MPs and the free MG in different dissolution media.

The saturated solubility of the free MG and the MG MPs in different dissolution media at 37 °C is displayed in Figure 6. From the Figure 6, the saturated solubility of the MG MPs and the free MG in water was about 641 μg/mL and 146 μg/mL, respectively, and the saturated solubility of the MG MPs was about 4.39 times that of the free MG; In AGJ, the saturated solubility of the MG MPs and the free MG was about 206 μg/mL and 98 μg/mL, respectively; and the saturated solubility of the MG MPs was about 2.1 times that of the free MG; In AIJ, the saturated solubility of the MG MPs and the free MG was about 119 μg/mL and 53 μg/mL, respectively, and the saturated solubility of the MG MPs was about 2.25 times that of the free MG. It can be seen from the above results that the saturated solubility of the MG MPs in three dissolution media was significantly higher than that of the free MG, which illustrated that reducing the particle size and increasing the specific surface area of the MG could be conducive to increase its solubility. Therefore, at the same dose, the MG MPs could better absorb, release and exert its efficacy in vivo than the free MG.

#### 3.4.2. In Vitro Dissolution Results

**Figure 7:**
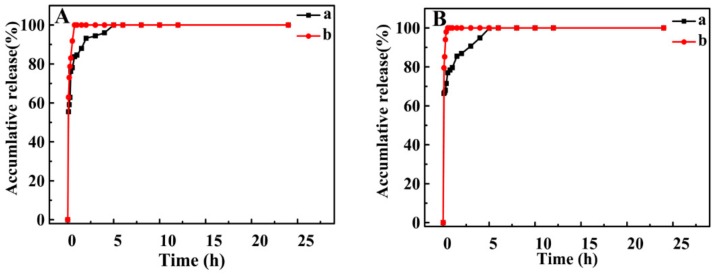
The dissolution profiles of the free MG (a) and the MG MPs (b) in artificial gastric juice (AGJ) (**A**) and artificial intestinal juice (AIJ) (**B**).

Furthermore, the MG MPs significantly improved drug dissolution compared to the free MG. The dissolution curves of the MG MPs (a) and the free MG (b) in AGJ and AIJ are shown in Figure 7. In the AGJ (Figure 7A), the maximum dissolution of the MG MPs reached 100% at approximately 0.75 h. However, only 83.75% of the free MG dissolved during the same period. In addition, in the AIJ (Figure 7B), the MG MPs achieving the maximum dissolution was approximately 100% at approximately 0.5 h. The dissolution of the free MG was approximately 76.96%. It can be seen that the dissolution rate of the MG MPs was obviously faster than that of the free MG, and was 1.19 times and 1.30 times that of free MG in AGJ and AIJ, respectively. Therefore, the MG MPs prepared was beneficial to improve the solubility and the dissolution of the MG by the SAS method.

### 3.5. Antioxidant Activity In Vitro

#### 3.5.1. DPPH Radical-Scavenging Activity Measurement.

**Figure 8:**
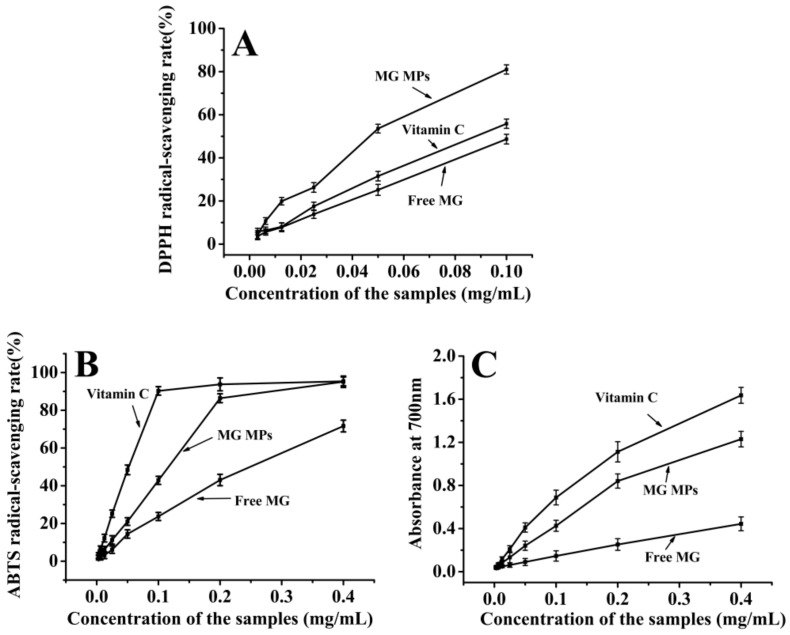
The 1,1-diphenyl-2-dinitrophenylhydrazine (DPPH) radical-scavenging activity (**A**), the ABTS radical-scavenging activity (**B**) and the reducing power (**C**) of the MG MPs, the free MG and the vitamin C.

The test of the DPPH radical-scavenging ability is an important method to investigate the antioxidant capacity of the drugs. As shown in Figure 8A, the DPPH radical-scavenging activities of all the samples increased with the concentrations. When the concentration of the samples was 0.025 mg/mL, the scavenging efficiency of vitamin C as control group was about 17.51%, the scavenging efficiency of the free MG was about 13.87%, while the scavenging efficiency of the MG MPs was about 26.31%; when the concentration of the samples reached 0.1 mg/mL, the scavenging efficiency of vitamin C was about 55.84%, the scavenging efficiency of the free MG was about 48.71%. However, the scavenging efficiency of the MG MPs was about 81.01% at the same concentration, which was about 1.66 times of the free MG and 1.45 times of vitamin C. In addition, from the half inhibition concentrations (IC_50_) of the samples, the IC_50_ of the vitamin C, the free MG and the MG MPs was about 0.114 mg/mL, 0.148 mg/mL and 0.040 mg/mL, respectively, which showed that the MG MPs had a higher DPPH radical-scavenging activity by lower IC_50_ than the free MG and the vitamin C.

#### 3.5.2. ABTS Radical-Scavenging Activity Measurement.

The test of ABTS radical-scavenging ability is another method to investigate the antioxidant capacity of the drugs. As shown in Figure 8B, with the increase of the concentration from 0.00313 to 0.4 mg/mL, the ABTS radical-scavenging ability of each sample increased gradually. When the concentration of the samples was 0.1 mg/mL, the scavenging efficiency of vitamin C as control group was about 90.29%, the scavenging efficiency of the free MG was about 23.72%, while the scavenging efficiency of the MG MPs was about 42.85%; when the concentration of the samples reached 0.4 mg/mL, the scavenging efficiency of vitamin C was about 95.36%, the scavenging efficiency of the free MG was about 71.67%. While, the scavenging efficiency of the MG MPs was about 95.12% at the same concentration, which was close to that of vitamin C, about 1.33 times of the free MG. Additionally, the IC50 of the vitamin C, the free MG and the MG MPs was about 0.0386 mg/mL, 0.245 mg/mL and 0.0763 mg/mL, respectively, which showed that the MG MPs had a higher ABTS radical-scavenging activity by lower IC50 than the free MG, which was consistent with the experimental results of the DPPH radical-scavenging ability.

#### 3.5.3. Reducing Power Measurement.

The reducing powers of the MG MPs, the free MG and the vitamin C was displayed in Figure 8C. It could be seen from the figure that the reducing power of each sample increased gradually with the concentration increasing from 0.00313 to 0.4 mg/mL. When the concentration was 0.1 mg/mL, the absorbance of the vitamin C as the control group at 700 nm was about 0.688, the absorbance of the free MG at 700 nm was about 0.146, and the absorbance of the MG MPs at 700 nm was about 0.426; when the concentration reached 0.4 mg/mL, the absorbance of vitamin C was about 1.636, the absorbance of the free MG was about 0.444, and the absorbance of the MG MPs was about 1.23. It could be seen from the above results that the reduction power of the MG MPs was significantly higher than that of the free MG. These results were also consistent with those of the DPPH and the ABTS radical-scavenging abilities. Therefore, the MG MPs had a stronger antioxidant capacity in vitro compared with the free MG, which was expected to become a new antioxidant drug formulation and produce a better response for clinical applications of MG.

### 3.6. Pharmacokinetic Analysis and Bioavailability

**Figure 9:**
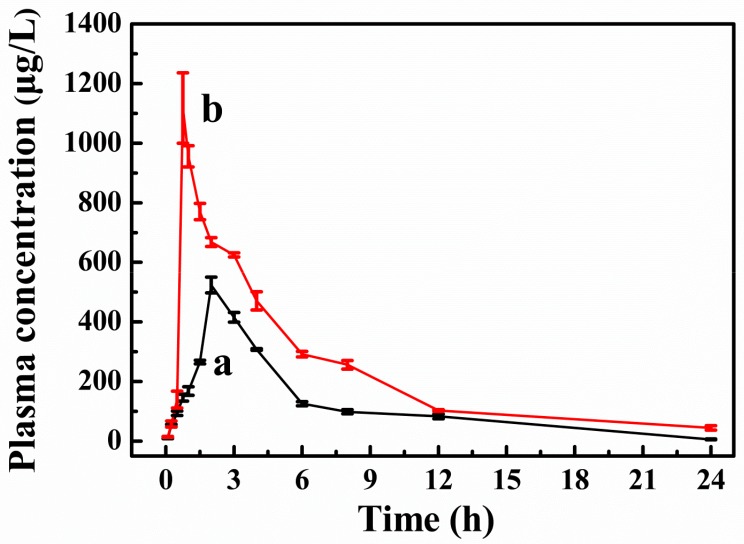
The plasma concentration–time profiles of the free MG (a) and the MG MPs (b).

After oral administration, the mean plasma concentration-time profiles for the MG MPs and the free MG are presented in Figure 9. The results demonstrated that both the MG MPs and the free MG concentration–time curves could be fitted to the two-compartment model. The relevant pharmacokinetic parameters for the compartmental analysis are listed in Table 4. As shown in Figure 9 and Table 4, the plasma concentration of rats treated with the MG MPs was always higher than that of the rats treated with the free MG at the same dosage. The plasma concentration of the MG reached the maximum concentration (C_max_) of 1084.580 μg/L after oral administration of the MG MPs, which was obviously increased 2.07-fold over that of the free MG (523.871 μg/L). The T_max_ (0.75 h) in rats treated with the MG MPs was also significantly shorter than that treated with the free MG (2 h). Moreover, the AUC(0–t) value of the MG MPs (10,163.112 μg/L*h) was significantly increased 4.644-fold than that of the free MG (2188.375 μg/L*h). Consequently, the MG MPs prepared in this study was highly effective in improving the rate and extent of absorption of MG, and was expected to become a new oral drug formulation with high bioavailability.

## 4. Conclusions

In this study, the MG MPs with high solubility and bioavailability have been successfully prepared by the SAS technology. Several main factors that have the effects on saturated solubility of the MG MPs have been evaluated by the orthogonal design (OAD) and the optimum conditions obtained were as follows: the precipitation pressure 20 MPa, the precipitation temperature 51 °C, the MG concentration 23 mg/mL, and the feeding speed 10 mL/min. Under the optimum conditions, the MG MPs with an average diameter of 532.8 nm was obtained, and the yield of the powders was about 95.3%. The solubility of the MG MPs was higher than that of the free MG, and was about 4.26, 2.1 and 2.5 times of that of the free MG in water, AGJ and AIJ, respectively. The dissolution rate of the MG MPs were also obviously higher than that of the free MG, and was about 1.19 and 1.30 times than that of the free MG in AGJ and AIJ. Additionally, the oral bioavailability of the MG MPs in rats was about 2.07 times higher than that of the free MG, and the MG MPs had a higher antioxidant capacity by lower IC50 than that of the free MG. Thus, the MG MPs prepared in this study may have great potential value to become a new oral MG formulation and possess potential clinical application value.

## Figures and Tables

**Table 1:** The factors and levels of the orthogonal array design.

Trial No.	(A) Precipitation Pressure (MPa)	(B) Precipitation Temperature (°C)	(C) MG Concentration (mg/mL)	(D) Feeding Speed (mL/min)
1	10	35	5	4
2	15	43	23	7
3	20	51	41	10
4	25	59	59	13

**Table 2:** Orthogonal array design (OAD) L_16_ (4^5^) and experimental results.

Trial No.	(A) Precipitation Pressure (MPa)	(B) Feeding Speed (mL/min)	(C) Precipitation Temperature (°C)	(D) MG Concentration (mg/mL)	Average Particle Size (nm)	Variation Coefficient (%)	Saturated Solubility (mg/mL)
1	1	1	1	1	981.2	5.4	0.2512
2	1	2	2	2	809.6	2.94	0.4293
3	1	3	3	3	762.5	3.96	0.4584
4	1	4	4	4	870.6	4.6	0.3504
5	2	1	2	3	832.3	5.4	0.4027
6	2	2	1	4	896.5	3.1	0.3303
7	2	3	4	1	813.2	2.39	0.4237
8	2	4	3	2	750.4	2.1	0.4627
9	3	1	3	4	590.9	2.9	0.5602
10	3	2	4	3	560.8	3.4	0.5897
11	3	3	1	2	667.4	3.38	0.5152
12	3	4	2	1	532.8	2.01	0.6013
13	4	1	4	2	1044.6	8.79	0.1940
14	4	2	3	1	938.4	7.24	0.2913
15	4	3	2	4	864.4	6.8	0.3659
16	4	4	1	3	922.4	5.34	0.2994
K_1_	0.372	0.352	0.349	0.392			
K_2_	0.405	0.410	0.450	0.400			
K_3_	0.567	0.441	0.443	0.438			
K_4_	0.288	0.428	0.389	0.402			
R ^b^	0.279	0.089	0.101	0.046			

**Table 3:** ANOVA analysis of four parameters for SAS micronization of mangiferin (MG).

Source	Sum of Squares (SS)	Degrees of Freedom (df)	F-ratio	F_0.10_	Type of Effect
(A) Precipitation pressure (MPa)	0.164	3	41.000	9.28	Significant
Feeding speed (mL/min)	0.019	3	4.750	9.28	
(C) Precipitation temperature (°C)	0.027	3	6.750	9.28	
(D) MG concentration (mg/mL)	0.005	3	1.250	9.28	

**Table 4:** Pharmacokinetic parameters for MG in rats after oral administration of the free MG and the MG MPs.

Pharmacokinetic Parameters	Free MG	MG MPs
C_max_ (μg/L)	523.871	1084.580
T_max_ (h)	2.000	0.750
t_1/2_ (h)	4.013	6.515
MRT(0–t) (h)	6.063	6.137
MRT(0–∞) (h)	6.348	8.114
AUC(0–t) (μg/L*h)	2188.375	10,163.112
AUC(0–∞) (μg/L*h)	2223.245	14,151.415

## References

[1] Khurana R.K., Gaspar B.L., Welsby G., Katare O.P., Singh K.K., Singh B. (2018). Improving the biopharmaceutical attributes of mangiferin using vitamin E-TPGS co-loaded self-assembled phosholipidic nano-mixed micellar systems. Drug Deliv. Transl. Res..

[2] Ma H., Chen H., Sun L., Tong L., Zhang T. (2014). Improving permeability and oral absorption of mangiferin by phospholipid complexation. Fitoterapia.

[3] Muruganandan S., Gupta S., Kataria M., Lal J., Gupta P.K. (2002). Mangiferin protects the streptozotocin-induced oxidative damage to cardiac and renal tissues in rats. Toxicology.

[4] Hou J., Zheng D., Fung G., Deng H., Chen L., Liang J., Jiang Y., Hu Y. (2015). Mangiferin suppressed advanced glycation end products (AGEs) through NF-κB deactivation and displayed anti-inflammatory effects in streptozotocin and high fat diet-diabetic cardiomyopathy rats. Can. J. Physiol. Pharmacol..

[5] Suchal K., Malik S., Gamad N., Malhotra R.K., Goyal S.N., Ojha S., Kumari S., Bhatia J., Arya D.S. (2016). Mangiferin protect myocardial insults through modulation of MAPK/TGF-β pathways. Eur. J. Pharmacol..

[6] Sánchez G.M., Re L., Giuliani A., Núez-Sellés A.J., Davison G.P., León-Fernández O.S. (2000). Protective effects of Mangifera indica L. extract, mangiferin and selected antioxidants against TPA-induced biomolecules oxidation and peritoneal macrophage activation in mice. Pharmacol. Res..

[7] Muruganandan S., Srinivasan K., Gupta S., Gupta P.K., Lal J. (2005). Effect of mangiferin on hyperglycemia and atherogenicity in streptozotocin diabetic rats. J. Ethnopharmacol..

[8] Wang B., Wan J., Gong X., Kuang G., Cheng X., Min S. (2015). Mangiferin attenuates renal ischemia-reperfusion injury by inhibiting inflammation and inducing adenosine production. Int. Immunopharmacol..

[9] Li X.J., Du Z.C., Huang Y., Liu B.M., Hu W.J., Lu W.J., Deng J.G. (2013). Synthesis and hypoglycemic activity of esterified-derivatives of mangiferin. Chin. J. Nat. Med..

[10] Yuan Y.F., Deng J.G. (2008). Preparation of mengoferin monosodium salt. Chin. J. Hosp. Pharm..

[11] Liao H.L., Qiu-Ye W.U., Hong-Gang H.U., Zang Z.H., Song L., Yang Q. (2008). Structure modification of mangiferin. West China J. Pharm. Sci..

[12] Pleguezuelos-Villa M., Nácher A., Hernández M.J., Ofelia Vila Buso M.A., Ruiz Sauri A., Díez-Sales O. (2019). Mangiferin nanoemulsions in treatment of inflammatory disorders and skin regeneration. Int. J. Pharm..

[13] Xuan X.Y., Wang Y.J., Tian H., Pi J.X., Zhang W.L. (2012). Study on prescription of self-microemulsifying drug delivery system of Mangiferin phospholipid complex. J. Chin. Med. Mater..

[14] Bhattacharyya S., Ahmmed S.M., Saha B.P., Mukherjee P.K. (2014). Soya phospholipid complex of mangiferin enhances its hepatoprotectivity by improving its bioavailability and pharmacokinetics. J. Sci. Food Agric..

[15] Zhou H., Han Y.M., Zheng Y.M., Xiu-Ying X.U., Shan-Quan F.U., Wang L.L., Zeng P.T. (2009). Preparative Procedure of Inclusion Compound of Mangiferin-HP-β-CD. J. Chongqing Institute of Technol..

[16] Mao X., Liu L., Cheng L., Cheng R., Zhang L., Deng L., Sun X., Zhang Y., Sarmento B., Cui W. (2019). Adhesive nanoparticles with inflammation regulation for promoting skin flap regeneration. J. Control. Release.

[17] Liu R., Liu Z., Zhang C., Zhang B. (2012). Nanostructured lipid carriers as novel ophthalmic delivery system for mangiferin: Improving in vivo ocular bioavailability. J. Pharm. Sci..

[18] Lai Y.L., Wang C.H., Smith K.A. (2008). Supercritical antisolvent production of biodegradable micro- and nanoparticles for controlled delivery of paclitaxel. J. Control. Release.

[19] Chattopadhyay P., Gupta R.B. (2001). Production of griseofulvin nanoparticles using supercritical CO2 antisolvent with enhanced mass transfer. Int. J. Pharm..

[20] Sun Z., Ma C.H., Yang L., Zu Y.G., Zhang R.R. (2011). Production of Ursolic Acid Nanoparticles by Supercritical Antisolvent Precipitation. Adv. Mater. Res..

[21] Kim M.S., Jin S.J., Kim J.S., Park H.J., Song H.S., Neubert R.H.H., Hwang S.J. (2008). Preparation, characterization and in vivo evaluation of amorphous atorvastatin calcium nanoparticles using supercritical antisolvent (SAS) process. Eur. J. Pharm. Biopharm..

[22] Montes A., Wehner L., Pereyra C., de la Ossa E.J.M. (2016). Mangiferin Nanoparticles Precipitation by Supercritical Antisolvent Process. J. Supercrit. Fluids.

[23] Sharma O.P., Bhat T.K. (2009). DPPH antioxidant assay revisited. Food Chem..

[24] Park J., Rho S.J., Kim Y.R. (2019). Enhancing antioxidant and antimicrobial activity of carnosic acid in rosemary (Rosmarinus officinalis L.) extract by complexation with cyclic glucans. Food Chem..

[25] Zhou Y., Ma W., Wang L., Sun W., Li M., Zhang W., Liu Y., Song X., Fan Y. (2019). Characterization and antioxidant activity of the oligo-maltose fraction from Polygonum Cillinerve. Carbohydr. Polym..

